# Developing a New Generation of Therapeutic Dental Polymers to Inhibit Oral Biofilms and Protect Teeth

**DOI:** 10.3390/ma11091747

**Published:** 2018-09-17

**Authors:** Ke Zhang, Bashayer Baras, Christopher D. Lynch, Michael D. Weir, Mary Anne S. Melo, Yuncong Li, Mark A. Reynolds, Yuxing Bai, Lin Wang, Suping Wang, Hockin H. K. Xu

**Affiliations:** 1 Department of Orthodontics, School of Stomatology, Capital Medical University, Beijing 100069, China; tuzizhangke@163.com; 2 Department of Advanced Oral Sciences and Therapeutics, University of Maryland Dental School, Baltimore, MD 21201, USA; bbaras@umaryland.edu (B.B.); mweir@umaryland.edu (M.D.W.); mmelo@umaryland.edu (M.A.S.M.); mreynolds@umaryland.edu (M.A.R.); hxu2@umaryland.edu (H.H.K.X.); 3 Restorative Dentistry, University Dental School and Hospital, University College Cork, Wilton T12 E8YV, Ireland; chris.lynch@ucc.ie; 4 Clinical Research Center of Shaanxi Province for Dental and Maxillofacial Diseases, Key Laboratory of Shaanxi Province for Craniofacial Precision Medicine Research, College of Stomatology, Xi’an Jiaotong University, Xi’an 710004, China; yuncong1982@mail.xjtu.edu.cn; 5 Department of Oral Implantology, School of Dentistry, Jilin University, Changchun 130012, China; 6 Department of Operative Dentistry and Endodontics & Stomatology Center, The First Affiliated Medical School of Zhengzhou University, Zhengzhou 450052, China; 7 Center for Stem Cell Biology & Regenerative Medicine, University of Maryland School of Medicine, Baltimore, MD 21201, USA; 8 University of Maryland Marlene and Stewart Greenebaum Cancer Center, University of Maryland School of Medicine, Baltimore, MD 21201, USA

**Keywords:** polymeric composites, bonding agents, antibacterial, oral biofilms, periodontal pathogens, caries inhibition

## Abstract

Polymeric tooth-colored restorations are increasingly popular in dentistry. However, restoration failures remain a major challenge, and more than 50% of all operative work was devoted to removing and replacing the failed restorations. This is a heavy burden, with the expense for restoring dental cavities in the U.S. exceeding $46 billion annually. In addition, the need is increasing dramatically as the population ages with increasing tooth retention in seniors. Traditional materials for cavity restorations are usually bioinert and replace the decayed tooth volumes. This article reviews cutting-edge research on the synthesis and evaluation of a new generation of bioactive dental polymers that not only restore the decayed tooth structures, but also have therapeutic functions. These materials include polymeric composites and bonding agents for tooth cavity restorations that inhibit saliva-based microcosm biofilms, bioactive resins for tooth root caries treatments, polymers that can suppress periodontal pathogens, and root canal sealers that can kill endodontic biofilms. These novel compositions substantially inhibit biofilm growth, greatly reduce acid production and polysaccharide synthesis of biofilms, and reduce biofilm colony-forming units by three to four orders of magnitude. This new class of bioactive and therapeutic polymeric materials is promising to inhibit tooth decay, suppress recurrent caries, control oral biofilms and acid production, protect the periodontium, and heal endodontic infections.

## 1. Introduction

Tooth caries is a widespread problem in the world. More than half of all dental restorations fail within 10 years, and recurrent (secondary) caries is a main reason for failures [1,2,3]. Replacing the failed restorations accounts for 50–70% of all tooth cavity restorations performed [4]. This represents a large economic burden; for example, the annual expense for restoring tooth cavities in the U.S. was $46 billion in 2005 [5]. In addition, the expense is rapidly climbing because of an aging population with longer life expectancies, and seniors are retaining more and more of their natural teeth [6]. Tooth-colored polymeric composites and bonding agents are the primary materials for restoring tooth cavities [3,7,8,9,10,11,12]. This is because advances in polymer chemistry and filler particle compositions have enhanced the composite restoration properties [13,14,15,16,17,18]. However, one key disadvantage is that polymeric composite materials tend to accumulate more oral biofilms than other dental materials such as metals and ceramics [19]. Oral biofilms ferment carbohydrates and produce acids that can lead to dental caries [20,21]. Therefore, researchers have devoted effort to synthesizing new antibacterial polymers for dental applications [22,23,24,25,26,27]. In general, antibacterial dental resins and composites can be divided into two classes. Class 1 uses polymerizable quaternary ammonium methacrylates (QAMs) where the antibacterial agent is bonded to be part of the polymer network. Class 2 uses filler particles with antibacterial activities that are filled into the polymer matrix. For class 1, QAMs were developed and incorporated into dental polymeric materials [22,23]. The first such material, 12-methacryloyloxydodecyl-pyridinium bromide (MDPB), was copolymerized with dental polymers and provided potent anti-biofilm effects [22,23]. Since then, other antibacterial resins were also synthesized and exhibited the capability to hinder bacterial growth and biofilm formation [25,26,28,29,30,31,32,33,34,35,36]. For Class 2, antibacterial fillers such as silver, zinc oxide and bioglass particles were mixed into polymer matrices, in which the antibacterial effect was achieved by the release of the agents [37,38,39,40,41,42,43]. While some studies reported sustainable long-term release of ions to exert antibacterial effects [44], other studies showed that the release and antibacterial efficacy decreased with increasing time [42,43]. Controlled long-term release of antibacterial agents has great potential for dental applications to combat caries and oral pathogens, especially via the use of nanotechnology and recharge and re-release mechanisms. This article focuses on Class 1 and reviews innovative developments in QAM-containing dental polymers and their exciting potential in restorative, preventive, root caries, periodontal, and endodontic applications.

## 2. Antibacterial Polymeric Dental Composites

Novel antibacterial polymeric composites were synthesized with functions to reduce oral biofilm acids and dental caries formation [22,23]. Antibacterial monomer MDPB was copolymerized into a resin composite which substantially reduced the glucan synthesis by *Streptococcus mutans* (*S. mutans*), a major cariogenic species, on the composite surface [45,46]. This was achieved without negatively influencing the composite’s mechanical properties and degree of polymerization conversion. A separate study synthesized polymeric composites with antibacterial and fluoride-releasing properties, which caused a large decrease in *S. mutans* biofilm formation [26]. Another study synthesized novel nanoparticles of quaternary ammonium polyethylenimine (QPEI) and incorporated them into a polymeric composite [47]. The QPEI composite resulted in a strong anti-biofilm activity in human participants in vivo against oral salivary bacteria [47]. In another study, researchers developed a furanone-containing composite with antibacterial functions, achieving a 16%–68% decrease in the viability of *S. mutans* grown on the composite surface [48]. 

**Figure 1:**
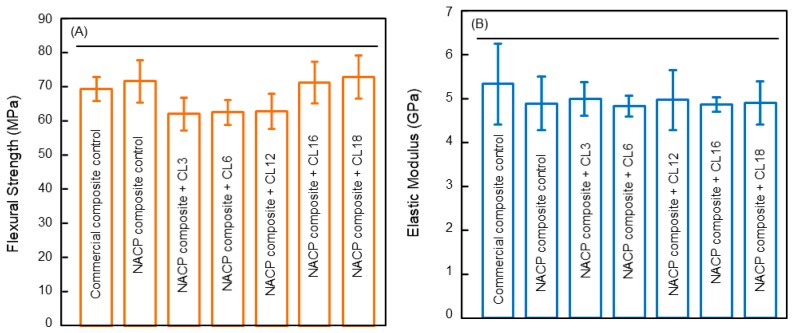
Mechanical properties of dental polymeric composites: (**A**) flexural strength, and (**B**) elastic modulus (mean ± SD; *n* = 6). Adding QAMs with amine alkyl chain length (CL) from 3 to 18 produced no significant loss in strength and elastic modulus. All QAM composites had mechanical properties similar to control composites without QAM. Horizontal line indicates *p* > 0.1. Adapted from [36], with permission from © 2015 Springer Nature.

Recently, a new class of QAMs with the alkyl chain length (CL) from 3 to 18 were developed and mixed into dental polymers to develop composites [36]. The QAMs were developed using a Menschutkin method in which a tertiary amine had reaction with an organo-halide [25,49]. Five QAMs with different CL values of 3 to 18 were produced. To fabricate a composite, the model polymer matrix was made of bisphenol A glycidyl dimethacrylate (BisGMA) and triethylene glycol dimethacrylate (TEGDMA) (Esstech, Essington, PA) which were mixed at 1:1 by weight, although the method was also applicable to other polymer matrices as well. To render the BisGMA-TEGDMA resin light-curable, camphorquinone (0.2%) and ethyl 4-*N*,*N*-dimethylaminobenzoate (0.8%) were added. This polymer matrix was denoted BT. To develop the composite, a filler level of 50% mass fraction of silanated glass filler particles (barium boroaluminosilicate glass, median particle size = 1.4 µm, Caulk/Dentsply, Milford, DE, USA) were incorporated for improving the mechanical properties to enable the composite in load-bearing restorations [36]. In addition, nanoparticles of amorphous calcium phosphate (NACP) were also mixed into the composite at a 20% mass fraction for the releases of calcium and phosphate ions and remineralization properties. Each QAM with each CL was incorporated into the composite at a 3% by weight [36]. The flexural strength and elastic modulus of the composite indicated that adding 3% QAM did not negatively compromise the mechanical properties (Figure 1A). All the QAM composites possessed mechanical properties similar to those of the composite without QAM and a commercial control composite without antibacterial properties [36].

**Figure 2:**
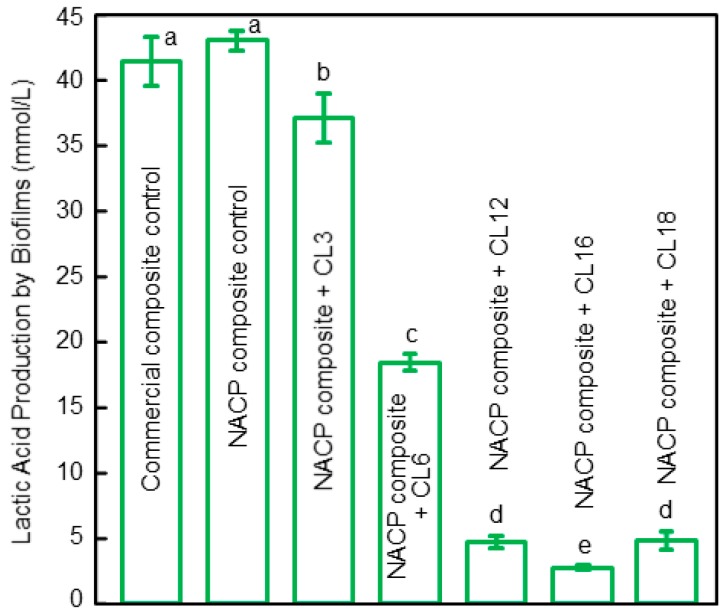
Lactic acid production by two-day dental plaque microcosm biofilms on the composites vs. QAM amine alkyl chain length (CL) (mean ± SD; *n* = 6). The polymeric composite using CL16 had the strongest anti-biofilm activity. Values indicated by dissimilar letters are statistically significantly different from each other (*p* < 0.05). Adapted from [36], with permission from © 2015 Springer Nature.

To test the antibacterial properties, saliva from human donors was used as an inoculum to obtain oral biofilms consisting of organisms from the mouth. This enabled the use of a dental plaque microcosm biofilm model [36]. Live/dead staining assay of two-day biofilms grown on the composite surface showed that increasing the CL of the QAM in the polymeric composite strengthened the antibacterial potency, which was the greatest at CL16. Raising the CL further to 18 reduced the antibacterial activity, compared to that of CL16. This was consistent with the lactic acid results from the biofilms on the surfaces of the composites (Figure 2) [36]. The two-day microcosm biofilms grown on the two control composite surfaces yielded the greatest amounts of lactic acid. Raising the CL from 3 to 16 substantially reduced the lactic acid production, reaching the minimum acid at CL16. Therefore, CL16 appeared to possess the strongest antibacterial activity among the groups tested. For the composite with CL16, the acid production of the adherent biofilms was reduced by an order of magnitude when compared with control composites. This acid reduction could contribute to reducing tooth mineral dissolution and caries occurrence [36].

Regarding the antibacterial mechanism, the QAM-incorporated polymer composite had quaternary amine N^+^ with positive charges which could interact with the cell membrane of the bacteria having negative charges. This could disrupt the membrane and cause cytoplasmic leakage, leading to bacterial death [30]. Other possible antibacterial mechanisms include preventing material transports across the bacterial cell membrane, interfering with signaling pathways or adhesive molecules at the bacterial wall, etc. It was suggested that quaternary ammonium materials with relatively long chains would be particularly effective with insertion into the bacterial membrane, thus inducing physical disruption to compromise the bacteria [22,23,30]. Indeed, a previous report on antibacterial glass ionomer materials showed greater antibacterial potency by using longer chain lengths [50]. Another study on three-dimensional biofilms also demonstrated that the oral biofilm thickness and the mass of biofilms were substantially reduced when the alkyl chain was raised from 3 to 16 [51]. These findings are in agreement with Figure 2 showing an increasing antibacterial potency for composites with increasing CL from 3 to 16, with CL16 being the most potent [36]. However, when CL was further increased to 18, the anti-biofilm potency was reduced. A possible explanation may be that when the alkyl chain becomes excessively long, the alkyl chain may be bent or curled. This would then contribute to the partial covering of the positively-charged quaternary ammonium groups, thus to some extent blocking the interactions electrostatically with the bacterial cells, and yielding a decrease in the anti-biofilm efficacy [34,36]. Another possible reason is that increasing the chain length leads to a larger thermal fluctuation amplitude that reduces the probability of these molecules penetrating into the outer bacterial membrane. Further study is needed to determine and understand the relationship between the quaternary amine chain length and the antibacterial potency. Meanwhile, tailoring and tuning of the polymeric compositions are needed to optimize the anti-biofilm, acid reduction, and mechanical and physical properties of dental composites.

## 3. Antibacterial Dental Bonding Agents

Bonding agents are used clinically to adhere the restoration to enamel and dentin, enabling the restoration to sustain repeated chewing forces in the oral environment without detachment. However, the weakest link of the restoration is the bonded composite-tooth interface, and its failure is the primary reason for the failure of the entire restoration. Therefore, extensive efforts were made to enhance the dentin bond strength and investigate the mechanisms of the tooth-restoration bond [7,52]. Studies indicated that it would be advantageous for the bonding agent to be antibacterial in order to suppress biofilm acids and avoid caries formation at the tooth–composite margins [22,23,28,29,31]. Studies suggested that antibacterial adhesives could help eradicate the residual bacteria inside the tooth cavity, as well kill the invading bacteria due to marginal leakage, which otherwise would allow the oral bacteria to invade into the tooth-restoration margins [28,29]. Indeed, previous studies demonstrated that dental adhesives with MDPB incorporation were able to kill *S. mutans* growth [23,53]. In addition, methacryloxyl ethyl cetyl dimethyl ammonium chloride (DMAE-CB) was synthesized and incorporated into adhesive to inhibit bacterial growth [54]. Furthermore, antibacterial primer containing MDPB was also developed, which demonstrated strong antibacterial functions [53]. In addition to MDPB, chlorhexidine (CHX) particles were mixed into a primer to obtain antibacterial properties [55]. Besides modifying commercial bonding agents with antibacterial agents, novel experimental bonding agents with antibacterial functions were also developed.

**Figure 3:**
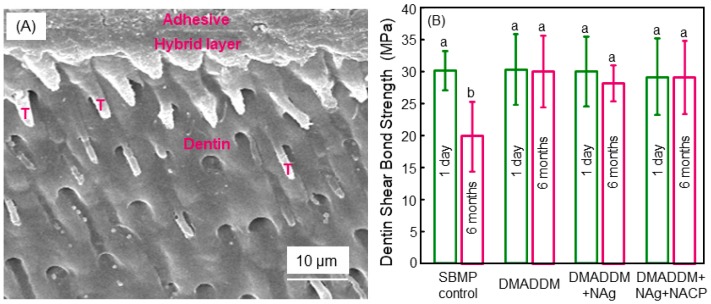
Dentin bonding. (**A**) Typical SEM image of the dentin–adhesive bonded region after one day of immersion. “T” indicates resin tags. (A) is for the DMADDM + Nag + NACP group; similar features were found in other groups. (**B**) Dentin bond strengths measured in shear (mean ± SD; *n* = 10). Values indicated by dissimilar letters are significantly different from each other (*p* < 0.05). Water-aging for six months caused a decrease of 35% in dentin bond strength for the commercial control bonding agent. In sharp contrast, the novel bioactive bonding agents containing DMADDM, NAg, and NACP showed no decrease in bond strength from one day to six months of water-aging. Adapted from [56], with permission from © 2013 Elsevier.

More recently, a therapeutic adhesive was synthesized that contained three agents: a QAM named dimethylaminododecyl methacrylate (DMADDM) with antibacterial activity, nanoparticles of silver (NAg), and NACP for remineralization [56]. This bonding agent showed a long-term durability in dentin bond strength. There was no reduction in dentin bond strength from one day to six months of immersion in water, while the commercial control bonding agent lost approximately one-third of its dentin bond strength at six months (Figure 3) [56]. Although many dental adhesives show satisfactory dentin bond strength in the short term, the long-term durability and stability of the resin–dentin interface remain a big challenge [57,58]. The resin–dentin bond strength demonstrated progressive decreases with increasing time in aging [59,60,61,62]. The reason for this decrease was attributed to the hydrolysis and enzymatic degradation of the exposed collagen and the adhesive resin, leading to the degradation of the hybrid layer at the dentin–adhesive interface [60]. There was water sorption in the aqueous oral environment because of the polar ether-linkages and the hydroxyl groups in the adhesive resin [61]. This could cause hydrolysis especially for the relatively more hydrophilic components in the resin [60,63]. Furthermore, the bacterial enzymes and the matrix metalloproteinases (MMPs) in the host tissues likely contributed significantly to the hybrid layer degradation [64]. During the dentin bonding, the MMPs were released and activated, which in turn could break down the collagen fibrils which became unprotected in the hybrid layer [64,65,66,67]. Such a damage of the collagen would in turn further increase the water sorption content, thus producing even more collagen degradation and causing deterioration in the dentin bonded interface [58]. In previous studies, CHX was shown to possess capabilities to inhibit the MMPs and suppress the enzymes [68]. Indeed, CHX was shown to nearly completely inhibit the collagen degradation of the demineralized dentin [69,70]. However, CHX can be dissolved in and cannot be co-polymerized with the resin, and, therefore, would be released in a relatively short amount of time, thus losing its long-term anti-MMP efficacy [24]. In contrast, DMADDM in Figure 3 was co-polymerized and immobilized in the polymer structure, and would not be leached out to diminish its effect over time, and hence could provide long-term MMP-inhibition [56]. Its durable anti-MMP effect likely contributed to maintaining the dentin bond strength without any decrease from one day to six months of water-aging treatment [56].

**Figure 4:**
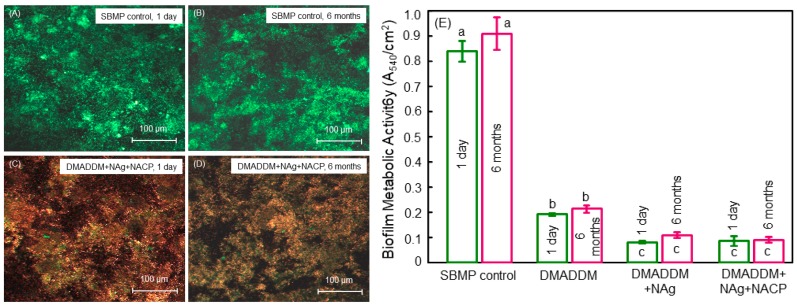
Anti-biofilm bonding resin after water-aging for 6 months. (**A**,**B**) Typical confocal laser scanning microscopy live/dead images for SBMP control; (**C**,**D**) DMADDM + Nag + NACP, at one day and six months, respectively. DMADDM and DMADDM + NACP groups had features similar to (**C**,**D**) (not shown). The novel bioactive bonding agent had primarily compromised bacteria. SBMP control had mostly live bacteria. (**E**) Metabolic activity (mean ± SD; *n* = 6). The potent antibacterial function remained after water-aging for six months. Adapted from Ref. [56], with permission from © 2013 Elsevier.

In addition, the bonding agent with DMADDM, NAg, and NACP incorporation possessed a strong antibacterial function with no decrease in the antibacterial potency from one day to six months of water-aging (Figure 4) [56]. This is consistent with the antibacterial agent being copolymerized and covalently bonded with the polymer network. This long-term antibacterial activity is beneficial considering that recurrent caries at the tooth-restoration margins is the primary cause for failures. By suppressing biofilm growth and reducing acids and enzymes, the antibacterial bonding agent could help suppress secondary dental caries. Furthermore, when the clinical requirements prevented the complete removal of the caries tissues such as avoiding the perforation of the pulp, as well as in minimal intervention dentistry [71], greater amounts of carious tissues were left in the tooth cavity. These carious tissues contained numerous residual bacteria inside the dentinal tubules in the prepared tooth cavity. The unpolymerized primer with the DMADDM anti-biofilm monomer, once applied to tooth cavity, would have direct contact with the tooth structure when flowing into the dentinal tubules, thereby eradicating the residual bacteria in the tubules. Then, upon polymerization, the adhesive resin at the margin would be in contact with the new invading bacteria, thus inhibiting their growth into the microgaps at the tooth-restoration interfaces [72]. While the six-month water-aging study indicated that the DMADDM copolymerization and covalent bonding with the polymer network enabled a long-term antibacterial activity, further longer-term study lasting for more than two years is needed on dentin bond strength, biofilm response, and caries prevention at the margins.

## 4. Antibacterial Composite for Tooth Root Caries Treatments

Senior people generally show greater risks of forming tooth root caries due to gingival recession and less saliva flow [73]. Periodontitis can lead to gingival recession, which in turn leads to more and more root surfaces to be exposed to the oral environment. Reduced saliva leads to more plaque buildup and less remineralization by saliva. These factors contribute to an increased risk of root caries. Root caries can be treated with Class V restorations. However, these restorations often have margins that are subgingival, which can provide pockets for bacterial growth that are difficult to clean, thus gradually leading to the loss of the periodontal attachment of the tooth. Indeed, it is a well-established knowledge that microbial biofilms are the primary etiological factor that causes periodontitis [74]. There are three primary species that are most often found in subgingival plaque from the periodontitis and periimplantitis areas: They are *Porphyromonas gingivalis* (*P. gingivalis*), *Prevotella intermedia* (*P. intermedia*), and *Aggregatibacter actinomycetemcomitans* (*A. actinomycetemcomitans*) [75]. In the periodontal pockets, these bacteria can generate virulence factors which lead to the gradual loss of the alveolar bone and the bone in periapical regions [75]. In areas with progressing periodontitis, *P. gingivalis* can serve as a keystone pathogen and as a portion of the climax group in the periodontal biofilms [76]. Being able to use estrogen and progesterone as an essential source of nutrients instead of using vitamin K, the *P. intermedia* species is connected with pregnancy gingivitis and periodontitis [77]. The third species, *A. actinomycetemcomitans*, is related to localized aggressive periodontitis. In addition to these three species, the fourth species, *Prevotella nigrescens* (*P. nigrescens*), is related to both healthy and diseased periodontium, and is biochemically comparable to *P. intermedia* [78]. In addition, *Fusobacterium nucleatum* (*F. nucleatum*), the fifth species, is linked to greater probing depth and periodontal ligament reduction [79]. Moreover, *F. nucleatum* can also promote the invasion of *P. gingivalis* into the gingival epithelial and aortic endothelial cells [80]. Last, *Enterococcus faecalis* (*E. faecalis*), the sixth species, is mainly considered an endodontic pathogen; however, it is also discovered in biofilms in the regions and in the saliva of patients who have chronic types of periodontal infections [81]. 

**Figure 5:**
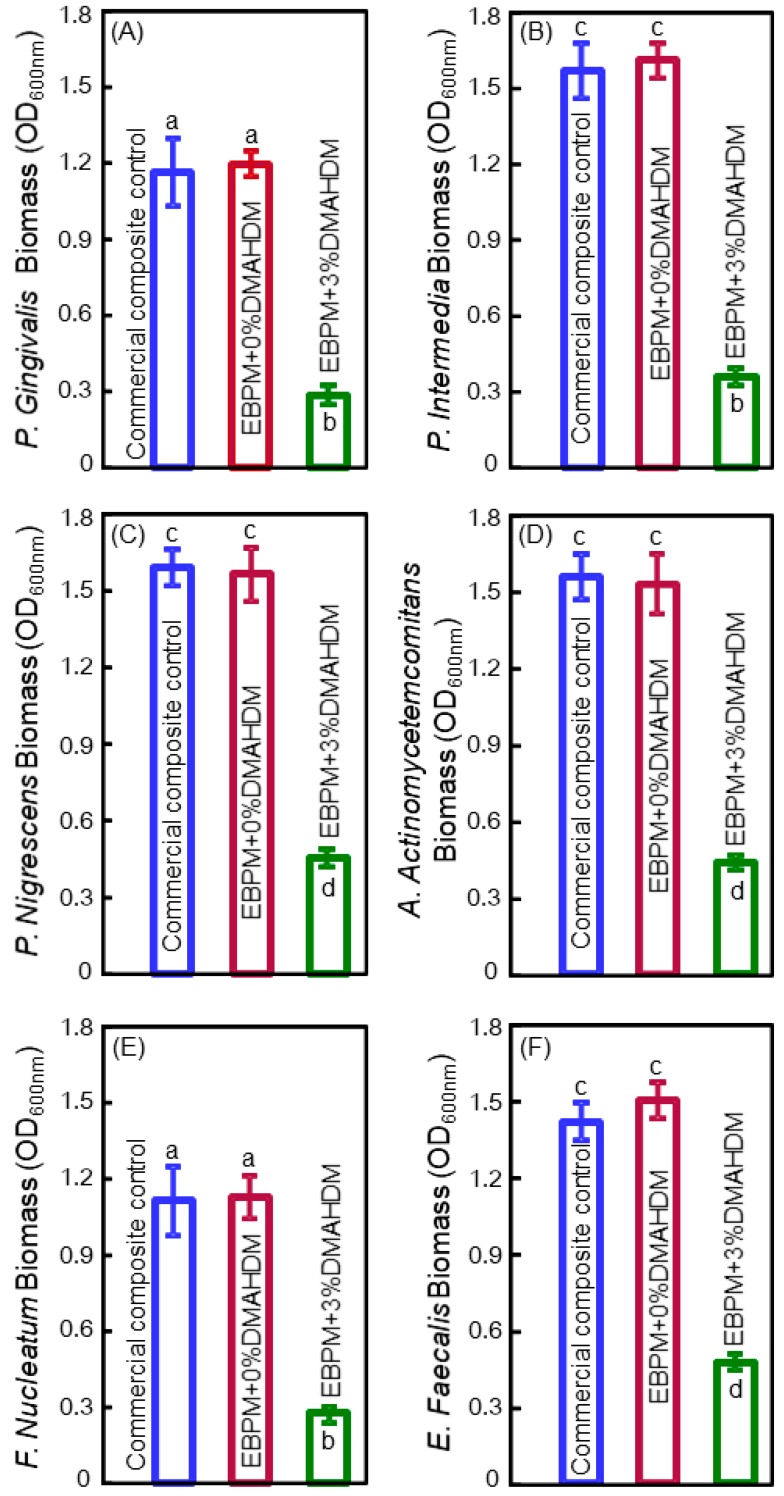
The biomass results of two-day biofilms grown on composites, evaluated using crystal violet assay and spectrophotometric optical density (OD600nm) (mean ± SD; *n* = 6): (**A**) *P. gingivalis*, (**B**) *P. intermedia*, (**C**) *P. nigrescens*, (**D**) *A. actinomycetemcomitans*, (**E**) *F. nucleatum* and (**F**) *E. faecalis*. The biofilm biomass on composites with DMAHDM was substantially reduced, as compared to that on composite without DMAHDM. Bars indicated by dissimilar letters are significantly different from each other (*p* < 0.05). Adapted from [82], with permission from © 2016 Elsevier.

Therefore, these six species were selected in a recent study [82]. That study reported a novel polymeric composite for Class-V tooth cavity restorations with therapeutic functions to combat the six types of pathogens related to the start and the exacerbation of periodontitis [82]. The polymer matrix consisted of ethoxylated bisphenol A dimethacrylate (EBPADMA) and pyromellitic glycerol dimethacrylate (PMGDM) at 1:1 mass ratio (referred to as EBPM). Dimethylaminohexadecyl methacrylate (DMAHDM) was added at 3% mass fraction into the composite. Disks of the polymeric composite were transferred to a new 24-well plate. Each type of bacteria was inoculated in 1.5 mL of medium at 10^7^ CFU/mL concentration in each well and cultured for 24 h. Then, the biofilm-disk constructs were transferred to new 24-well plates. New medium was added and the samples were cultured for another 24 h, thus totaling two days of culture to grow biofilms on the polymer surface [82]. Figure 5 shows the biofilm biomass after curing for two days which was measured using the absorbance values tested at OD_600nm_ [82]. The commercial control composite and the EBPM composite with 0% DMAHDM had similar biomass values. The EBPM composite with 3% DMAHDM had much less biofilm biomass. Therefore, the DMAHDM composite diminished the biomass of the biofilms for all six types of periodontitis-related pathogens [82].

**Figure 6:**
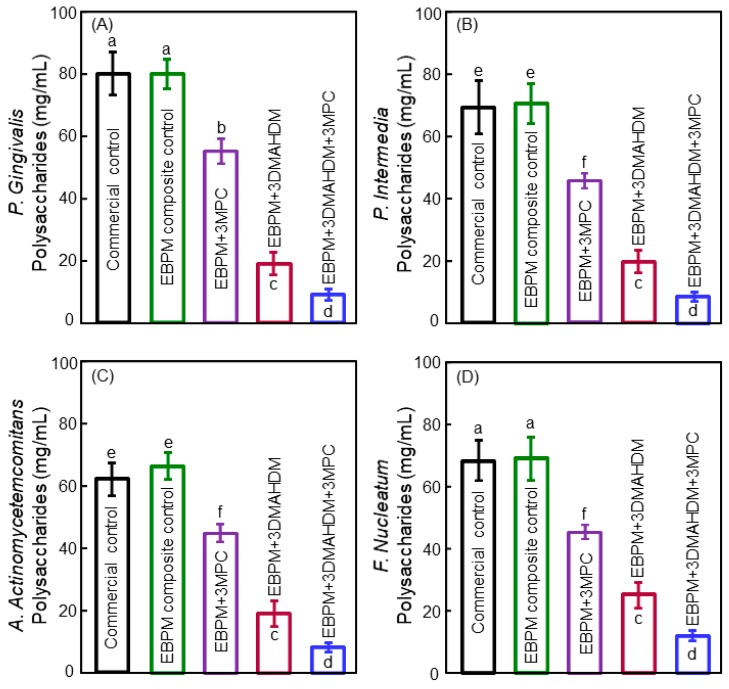
Polysaccharide results in biofilms grown for two days on polymeric composites: (**A**) *P. gingivalis*, (**B**) *P. intermedia*, (**C**) *A. actinomycetemcomitans*, and (**D**) *F. nucleatum* (mean ± SD; *n* = 6). Values indicated by dissimilar letters are significantly different (*p* < 0.05). Adapted from [83], with permission from © 2016 Elsevier.

In another study, protein-repellent agent 2-methacryloyloxyethyl phosphorylcholine (MPC) and antibacterial agent DMAHDM were combined in the polymeric composite to inhibit periodontal pathogens [83]. Figure 6 shows the polysaccharide amounts produced by the biofilms on the composites with: (A) *P. gingivalis*, (B) *P. intermedia*, (C) *A. actinomycetemcomitans*, and (D) *F. nucleatum* [83]. Biofilms on the commercial control composite and EBPM control composite produced similar quantities of polysaccharide. In contrast, biofilms on the EBPM + 3DMAHDM + 3MPC composite produced much less polysaccharide. Hence, the composite EBPM + 3DMAHDM + 3MPC could suppress periodontal pathogens and their production of the extracellular matrix [83]. Furthermore, the addition of MPC and DMAHDM into the polymeric composite did not adversely affect the mechanical properties. In addition, the use of dual agents of MPC + DMAHDM exerted a substantially more potent anti-biofilm activity, than using MPC or DMAHDM alone, against periodontal pathogens [83]. Therefore, the polymeric composite containing 3% DMAHDM and 3% MPC appeared to be the optimal composition. It showed a high potential for applications in Class-V tooth cavity restorations to inhibit periodontal biofilms, by reducing biofilm CFU by four orders of magnitude for all the types of periodontitis-related pathogens examined in that study [83].

## 5. Antibacterial Bonding Agents Inhibiting Periodontal Pathogens

Three bioactive agents (NACP for remineralization, MPC for protein-repellency, and DMAHDM for anti-biofilm activity) were combined into a polymeric bonding agent to suppress periodontal pathogens [84]. The adhesive contained PMGDM, EBPADMA, 2-hydroxyethyl methacrylate (HEMA) and BisGMA at 45/40/10/5 mass ratio (referred to as PEHB). The dentin shear bond strength results showed that adding 30% NACP into the adhesive did not compromise the dentin bond strength, compared to the control without NACP. In addition, incorporation of 5% DMAHDM + 5% MPC into both the primer and the adhesive did not negatively influence the dentin bond strength, compared to PEHB-NACP group without DMAHDM and MPC [84]. However, the incorporation of 5% DMAHDM + 7.5% MPC did lower the bond strengths. Therefore, a mass fraction of 30% NACP was incorporated into the adhesive, and mass fractions of 5% DMAHDM + 5% MPC were incorporated into both primer and adhesive [84]. 

**Figure 7:**
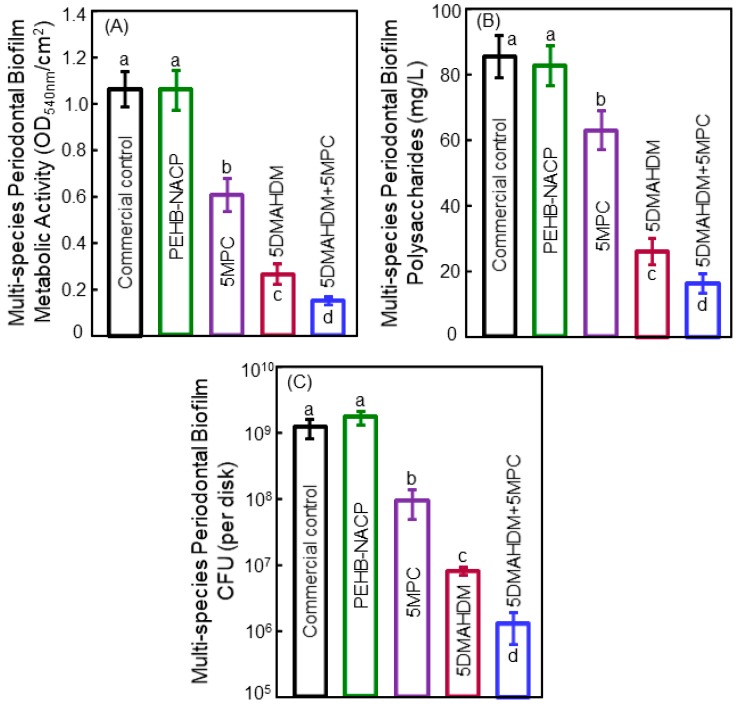
Multi-species periodontal biofilm: (**A**) metabolic activities, (**B**) polysaccharides production, and (**C**) CFU values on different dental bonding adhesive disks (mean ± SD; *n* = 6). The *y*-axis has the log scale in plot (**C**). Values indicated by dissimilar letters are significantly different from each other (*p* < 0.05). Adapted from [84], with permission from © 2017 RSC Advance.

Figure 7 shows the (A) metabolic activity, (B) polysaccharide, and (C) biofilm colony-forming units (CFU) for the multispecies periodontal biofilms [84]. The commercial bonding agent control and the PEHB-NACP without DMAHDM and MPC had similar CFU counts, indicating that NACP had little anti-biofilm activity. In contrast, DMAHDM or MPC each alone substantially decreased the biofilm CFU than those of the controls. Furthermore, the incorporation of 5% DMAHDM + 5% MPC resulted in the lowest metabolic activity, polysaccharide, and biofilm CFU counts. The CFU of the periodontal biofilm grown on the PEHB + 5DMAHDM + 5MPC polymer was three orders of magnitude less than that grown on the PEHB control polymer [84].

On a clean polymer surface in the oral environment, the saliva-derived proteins deposit on the polymer first, and then bacteria start to attach to the polymer. Salivary protein adsorption on the surface is required and is a prerequisite for oral bacteria adherence on the polymer surface [85]. This mechanism indicates that developing a protein-repellent polymer can greatly reduce biofilm growth on the polymer restoration in the oral environment. MPC is a methacrylate with phospholipid polar group in the side chain, with the capability to reduce protein adsorption and bacterial adhesion [34]. The mechanism for the protein-repellency was attributed to that in the hydrated MPC polymer, a large amount of free water exists around the phosphorylcholine groups which could detach the proteins [86]. Adding 5% of MPC into the bonding agent decreased the amount of protein adsorption by more than an order of magnitude [84]. In addition, combining the MPC with DMAHDM incorporation produced the strongest suppression of periodontal biofilms. The periodontal multi-species biofilm CFU was approximately 10^9^ counts on the control adhesive polymer. The CFU was decreased to 10^8^ counts by the use of MPC. The CFU was lowered to 10^7^ counts with the use of DMAHDM. In contrast, the CFU was reduced to only 10^6^ counts when both MPC and DMAHDM were used together in the bonding agent [84]. This synergistic reduction in biofilm growth on polymer surfaces was related to the mode of action, which was contact-inhibition [22,23]. When the cell membrane of the bacteria with negative charges contacts the quaternary amine N^+^ with positive charges on the polymer, the membrane could be disrupted, thus causing cytoplasmic leakage [30,47]. This mechanism of contact-inhibition implied that, when the polymer surface was covered by the salivary protein pellicles, the polymer surface was separated from the overlaying biofilm. This reduced the extent of contact, and hence the contact-inhibition efficacy was decreased. Therefore, because of the protein-repellency of the MPC, it helped diminish protein coverage on the polymer surface, thus exposing more polymer surface with quaternary amine N^+^ sites, thereby promoting the contact-inhibition ability. Therefore, the dual use of DMAHDM and MPC in the dental polymer could work synergistically to maximize the periodontal bacteria inhibition capability [84].

## 6. Antibacterial Polymeric Endodontic Sealers

Endodontic treatment is needed to eradicate bacterial infection in the tooth root canal, to avoid the microorganisms from harming the periapical healing and causing apical lesions [87]. Clinically, the anatomic complexity of the tooth root canal renders the complete debridement of bacteria practically impossible [88]. Such persistence of bacteria in the tooth root canal often results in post-treatment diseases [89]. One promising approach to address this challenge is the development of antibacterial root canal sealers with the capability to kill endodontic pathogens.

**Figure 8:**
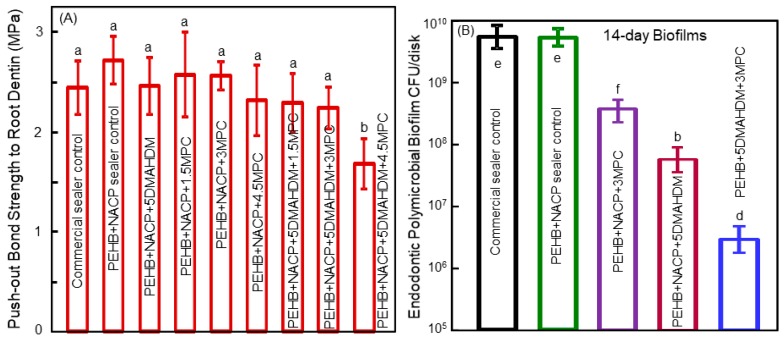
Polymeric endodontic sealers. (**A**) The push-out bond strength values to tooth root dentin (mean ± SD; *n* = 6). All the groups had similar strengths, except PEHB+NACP+5DMAHDM+4.5MPC which had a lower strength (*p* < 0.05). (**B**) The CFU of endodontic biofilm on the endodontic sealer grown for 14 days (mean ± SD; *n* = 6). In each plot, values with dissimilar letters are significantly different from each other (*p* < 0.05). Adapted from [90], with permission from © 2017 Elsevier.

A recent study developed a bioactive endodontic sealer with a good sealing ability in bonding to root dentin, indicated by a push-out strength being similar to those of commercial control without bioactive properties [90]. The push-out bond strength results to root wall dentin are shown in Figure 8A. The addition of 5% DMAHDM and 3% MPC into both the primer and the sealer paste did not adversely influence the dentin bond strength. However, when 5% DMAHDM and 4.5% MPC were incorporated together into the sealer, the push-out strength decreased. Hence, the composition of 5% DMAHDM and 3% MPC was determined to be optimal and was employed for the endodontic sealer and the primer [90]. Figure 8B shows the CFU results of the multispecies endodontic biofilms grown for 14 days on polymer samples. The commercial control group and the PEHB control polymer had similar CFU results. The addition of either DMAHDM or MPC alone reduced the endodontic biofilm CFU. The bioactive endodontic sealer containing 5% DMAHDM and 3% MPC had the lowest biofilm CFU. The 14-day endodontic biofilm CFU on the PEHB + NACP + 5DMAHDM + 3MPC polymer samples was three orders of magnitude less than that on the PEHB+NACP control polymer samples [90]. 

In a study on three-dimensional (3D) biofilms grown on dental polymer surfaces, the percentage of live bacteria was determined as a function of the location of the 2D cross-section inside the 3D biofilm at various distances from the polymer surface [91]. Near the surface of the polymer which contained DMAHDM, there were more dead bacteria in the biofilm. In the 3D biofilm away from the polymer surface, the percentage of live bacteria increased, likely due to a decrease in the contact-inhibition efficacy [91]. These results are consistent with the results of the DMAHDM-containing endodontic sealer, which achieved a greater reduction in the biofilm CFU at 3 days, compared to the reduction at 14 days [90]. The reason for the more killing effect at 3 days, and less killing effect at 14 days, was likely related to the contact-killing mechanism. The compromised bacteria on the polymer surface acted as a bridge for the further adherence and growth of bacteria, and the next layer of bacteria were away from the polymer surface with a reduced extent of contact-inhibition [92]. Therefore, the contact-inhibition mode of action would indicate that the antibacterial activity against the 14-day biofilms would be decreased because of a lack of direct contact when the microbes lived in the 3D biofilm structure away from the polymer surface. Therefore, the 14-day biofilm model represented a rigorous test of antibacterial activity. The fact that the PEHB + NACP + 5DMAHDM + 3MPC polymer was able to successfully kill and reduce the 14-day biofilm CFU by three orders of magnitude (Figure 8) indicates a novel bioactive endodontic sealer with an extremely potent anti-biofilm function [90]. Novel dental biomaterial development has the potential to bring tremendous benefits to treatment efficacy and improve the quality of life [7,8,9,10,11,12,13,14,15,16,17,93,94]. Further investigation is needed to achieve the long-lasting biofilm-eradication, therapeutic effects, and tooth-protection via the new bioactive dental polymeric materials using clinically-relevant experiments in the oral environment of human participants.

## 7. Conclusions

Currently available dental polymeric composites and bonding agents for tooth cavity restorations are usually bioinert. Since oral bacteria and biofilms play an important role in dental caries and oral infections, a new generation of dental polymeric materials are being developed that are bioactive and possess therapeutic effects including antibacterial, acid-reduction, protein-repellent, and remineralization capabilities. This article reviewed cutting-edge research on the development and properties of novel antibacterial dental polymeric composites, antibacterial bonding agents, bioactive root caries composites for senior patients, adhesives that can suppress periodontal pathogens, and antibacterial and protein-repellent endodontic sealers that can kill endodontic pathogens. Substantial reductions in oral biofilm metabolic activity, acid production, biomass, and polysaccharide synthesis were achieved with the tailored polymeric compositions. Biofilm CFU counts were reduced by three to four orders of magnitude. One advantage of QAM-containing polymers is that the antibacterial agent is co-polymerized and covalently bonded with the polymer, and hence it has long-term antibacterial function that is not leached out and lost over time. The disadvantage is that QAM-polymers rely on the contact-inhibition mechanism, with reduced antibacterial efficacy when the polymer surface is covered by a layer of salivary proteins. As alluded in the Introduction, one potential future development would be to combine strategies from Class 1 and Class 2 so that the dental polymer would possess long-term contact-inhibition as well as the release of antibacterial agents to inhibit bacteria away from the polymer surface throughout the three-dimensional biofilm. The advances in the anti-biofilm properties and therapeutic capabilities of the new generation of dental polymeric materials are expected to bring significant benefits to a wide range of restorative and preventive dental applications.
